# Does Radiotherapy for the Primary Tumor Benefit Prostate Cancer Patients with Distant Metastasis at Initial Diagnosis?

**DOI:** 10.1371/journal.pone.0147191

**Published:** 2016-01-25

**Authors:** Yeona Cho, Jee Suk Chang, Koon Ho Rha, Sung Joon Hong, Young Deuk Choi, Won Sik Ham, Jun Won Kim, Jaeho Cho

**Affiliations:** 1 Departments of Radiation Oncology, Yonsei University College of Medicine, Seoul, Korea; 2 Department of Urology, Yonsei University College of Medicine, Seoul, Korea; Dresden University of Technology, GERMANY

## Abstract

**Purpose/Objectives:**

Treatment of the primary tumor reportedly improves survival in several types of metastatic cancer. We herein evaluated the efficacy and toxicity of radiotherapy for the primary tumor in prostate cancer with metastasis.

**Materials/Methods:**

The study cohort included 140 men with metastatic prostate cancer at initial diagnosis. Metastatic sites were divided into 4 groups as follows: solitary bone, 2–4 bones, ≥5 bones, and visceral organs. Patient, tumor, and treatment characteristics, and clinical outcomes were compared between patients treated with (prostate radiotherapy [PRT] group) or without radiotherapy to the primary tumor.

**Results:**

Patients in PRT group presented with a statistically significantly younger age (*p* = .02), whereas other characteristics showed no significant difference. Overall survival (OS) and biochemical failure-free survival (BCFFS) were improved in PRT patients (3-year OS: 69% vs. 43%, *p* = 0.004; 3-year BCFFS: 52% vs. 16%, *p* = 0.002). Multivariate analysis identified PRT as a significant predictor of both OS (hazard ratio [HR] = 0.43, *p* = 0.015). None of the 38 PRT patients experienced severe (grade ≥3) genitourinary or gastrointestinal toxicity.

**Conclusions:**

Our data suggest that radiotherapy to the primary tumor was associated with improved OS and BCFFS in metastatic prostate cancer. The results of this study warrant prospective controlled clinical trials of this approach in stage IV prostate cancer patients with limited extent of bone metastasis and good performance status.

## Introduction

Aggressive treatment of the primary tumor is usually not recommended for patients with stage IV metastatic cancers. However, in certain types of malignancy, local treatment of the primary site with systemic therapy reportedly improves overall survival (OS) or enhances the efficacy of other therapeutics for the metastatic disease. In 1989, the Southwest Oncology Group initiated a randomized trial of nephrectomy in metastatic renal-cell cancer [1,2]. This study demonstrated that nephrectomy followed by interferon therapy resulted in longer survival among patients with metastatic renal-cell cancer than interferon therapy alone. In another trial, nephrectomy significantly improved the median OS from 7 to 17 months [3]. In 2004, Temple *et al*. reported that metastatic colon cancer patients undergoing primary tumor resection had a better response to chemotherapy [4]. Recent retrospective studies have shown that radiotherapy (RT) to or surgical excision of the primary tumor is associated with a better prognosis in patients with metastatic breast cancer [5,6]. Furthermore, the prognosis of stage IV metastatic diseases is relatively good in the above-mentioned cancer types. Even in advanced cancer, local treatment becomes more important as patient survival time increases because it can prevent subsequent distant seeding and relieve local symptoms due to progression of the primary site.

Similarly, prostate cancer patients with stage IV metastatic diseases also have a relatively good survival [7]. Hormonal therapy is the most commonly used first-line therapy for metastatic prostate cancer [8]. However, systemic therapy alone has resulted in a median survival of no more than 36 months in metastatic prostate cancer patients [9–11]. An intuitive argument is that in potentially metastatic cancer, the longer the primary tumor remains, the more metastatic events will occur [12]. Cytoreductive treatment via primary tumor radiotherapy may have a role in the management of metastatic prostate cancer.

Moreover, the systemic effect of RT, called the abscopal effect, has been explored in several studies [13,14]. The local inflammatory reactions generated by RT activate several immunological signals and contribute to better antigen cross-presentation leading to CD8+cytotoxic T-cell activation. In other words, the signals induced by RT could convert the irradiated tumor into an immunogenic antigen and the host’s immune system response to the tumor can contribute both to the local response to RT and to a systemic rejection of metastasis [14].

In general, local treatment for the primary tumor includes surgery and radiotherapy. For prostate cancer, radiation therapy is less invasive with newly developed techniques to minimize side effects [15]. If the therapy is well tolerated with minimal side effects, more aggressive treatment to the primary tumor can be applied in metastatic diseases.

In the present study, we aimed to investigate the role of radiotherapy to the primary tumor in patients with prostate cancer presenting as M1 diseases, with an assumption that the natural course of the diseases could be improved if the primary tumors are treated locally together with systemic therapy.

## Materials and Methods

### Patient eligibility

We identified 3,578 consecutive men who were diagnosed with prostate cancer between 2003 and 2011 at our institution. All men presented with metastatic disease at initial diagnosis or within 1 month from the initial diagnosis were included. Patients with other primary malignancies or those who underwent prostatectomy before radiotherapy were excluded. The final study cohort of this study included 140 men. The American Joint Committee on Cancer (AJCC) stage was used to classify metastatic disease. Metastases to the pelvic lymph node (LN), including the obturator, iliac, sacral, and hypogastric LN were defined to be regional LN metastases, and metastases beyond the pelvic cavity were classified as distant metastases, clinically M1 disease.

This study was approved by Institutional review board (IRB) of Yonsei University Health System (IRB protocol number 4-2014-0842). All data were collected by analyzing medical reports and information in our institutional medical records. However, due to the retrospective aspect of this study, patient informed consent was not provided for this study. But all subjects had provided written permission for their medical records to be used for research purposes as provided for.

### Treatment type and indication

**Fig 1:**
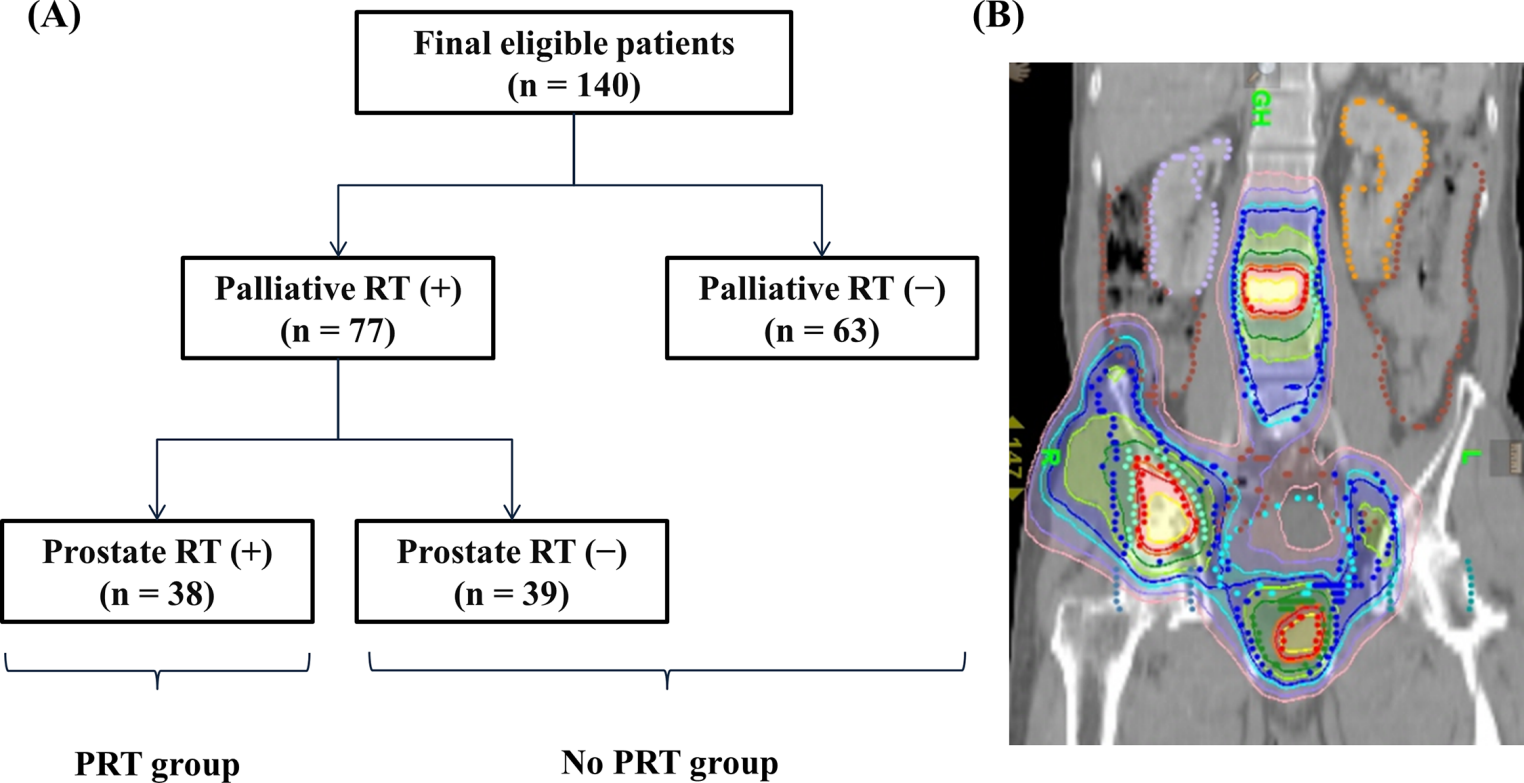
Treatment scheme of all patients (A) and an example of the treatment for a patient receiving prostate radiotherapy (B).

Of 140 eligible men in this study, 63 (45%) did not receive any radiotherapy. Among 77 patients (55%) who received radiotherapy, local radiotherapy to the primary tumor was performed in 38 patients (27%) (Fig 1A). In the present study, local treatment to the primary tumor was confined to radiotherapy delivered to the prostate, but not surgery. Radiotherapy was delivered to the prostate with pre-defined margins according to European Organisation for Research and Treatment of Cancer (EORTC) guideline [16], and metastatic lesions were treated to include gross tumor volume and appropriate margins (Fig 1B). Indication for radiotherapy to the prostate was decided based on the needs of patients with urinary symptoms and personalized at the discretion of the treating radiation oncologist. Systemic therapy, as indicated by the treating urologist or oncologist, might be given before radiotherapy and immediately after diagnosis. Indications for radiotherapy to metastatic lesions included pain, risk of fracture, and neurologic complications or disease control without any symptom. Most patients (96%) started androgen deprivation therapy at the time of initial diagnosis.

### Outcome and toxicity analysis

In this study, the primary endpoint was OS, and the secondary endpoints included biochemical failure-free survival (BCFFS). The Phoenix definition (nadir + 2 ng/mL) is commonly used due to several limitations of the previous ASTRO definition. However, the Phoenix definition also has limitations and a controversy still exists. We defined BCFFS as three consecutive increases in the prostate-specific antigen (PSA) level after nadir (ASTRO definition) for the following reasons. First, our patients had metastatic disease with more extensive lesions and their baseline PSA level was relatively higher (median 190 ng/mL, range 3.1–17800 ng/mL) than those without metastasis. Second, unless all tumors are cured, there are still residual tumors and the nadir PSA levels do not fall to subclinical levels (<4 ng/ml) in many patients. Third, patients treated with ADT frequently show PSA bounces [17] and a nadir + 2 ng/mL definition could result in more false-positive incidents in metastatic patients. In addition, the definition of castration-resistant prostate cancer is rising PSA levels or progressive disease in the setting of serum testosterone levels within the castrate range (< 50 ng/dl) [18]. Overall, considering these reasons, we thought the ASTRO definition is more applicable in metastatic cancer and we used the ASTRO definition in our study.

Toxicity monitoring was performed on patients receiving radiotherapy during all follow-up visits. Gastrointestinal (GI) or genitourinary (GU) toxicity was scored according to the Radiation Therapy Oncology Group and EORTC criteria. Hematologic toxicity was scored according to the Common Terminology Criteria for Adverse Events version 4.0. Each patient who received radiotherapy underwent weekly hematologic evaluation. The parameters included all hematologic abnormalities, GI or GU symptoms, and other adverse events reported by patients.

### Statistical analysis

Two groups of patients were analyzed in this study: those whose primary tumor was treated with radiotherapy (the prostate radiotherapy [PRT] group) and those who did not received radiotherapy to their primary tumor. Comparisons of patient and treatment characteristics between the two groups were performed using the chi-square and Fisher exact tests. The rates of OS and BCFFS were calculated by the Kaplan–Meier method. Statistical significance of survival differences was examined using the log-rank test. Cox’s regression model was used for multivariate analysis of OS. All significance was established at *p* < 0.05. All statistical analyses were performed using the SPSS software package, version 20.0.0 (SPSS Inc., Chicago, IL, USA).

## Results

### Patient and radiotherapy characteristics

Table 1 summarizes the clinical characteristics of the entire cohort and comparison between patients who received prostate radiotherapy and those who did not. The median age was 69 years, and 55% of the patients were ≥70 years. The Eastern Cooperative Oncology Group (ECOG) performance status was 0–1 in 79% of patients; 74% had a Gleason score ≥8; and the initial PSA levels were ≥100 ng/mL in 61% of patients. The number of metastatic lesions on initial presentation was 1 in 13% of patients, 2–4 in 30%, and ≥5 in 40%, with 78% of the cases being bone metastases. Age was the only factor with significant distribution difference between PRT and non-PRT patients, whereas no significant differences in the distribution of performance status, Gleason score at diagnosis, initial PSA level, disease extent, metastatic site, and the use and duration of androgen deprivation therapy were observed between the two groups.

**Table 1:** Characteristics of the entire patient cohort and comparisons between patients with and without prostate radiotherapy (PRT).

		Entire cohort	PRT (−)	PRT (+)	*p* value
		N = 140	N = 102	N = 38	
Characteristic		N (%)	N (%)	N (%)	
Age (years)	<70	77 (55)	50 (49)	27 (71)	*0*.*02*
	≥70	63 (45)	52 (51)	11 (29)	
ECOG status	0–1	111 (79)	77 (75)	34 (89)	*0*.*099*
	2–3	29 (21)	25 (25)	4 (11)	
Histology	Adenoca	136 (97)	98 (96)	38 (100)	*0*.*216*
	Others	4 (3)	4 (4)	0 (0)	
Type	Acinar	120 (86)	89 (87)	31 (82)	*0*.*427*
	Others	14 (10)	10 (10)	4 (11)	
	Unknown	6 (4)	3 (3)	3 (8)	
GS	≥8	104 (74)	79 (77)	25 (66)	*0*.*122*
	<8	22 (16)	16 (16)	6 (16)	
	Unknown	14 (10)	7 (7)	7 (18)	
Hormonal therapy	Yes	136 (97)	98 (96)	38 (100)	*0*.*213*
	No	4 (3)	4 (4)	0 (0)	
iPSA (ng/mL)	Median	191.5	221.0	126.0	*0*.*134*
	<100	54 (39)	36 (35)	18 (47)	*0*.*426*
	100–200	17 (12)	13 (13)	4 (11)	
	>200	69 (49)	53 (52)	16 (42)	
Number of metastases	1	25 (13)	13 (13)	12 (32)	*0*.*642*
on presentation	2–4	42 (30)	30 (29)	12 (32)	
	≥5	57 (40)	46 (45)	11 (29)	
Metastasis	Visceral + others	32 (23)	27 (26)	5 (15)	*0*.*126*
	Bone only	108 (77)	75 (74)	33 (85)	
Nadir PSA (ng/mL)	Median	1.11	1.12	0.61	*0*.*042*
Nadir PSA < 4 ng/mL		89 (64)	56 (55)	33 (87)	*0*.*02*

*Abbreviations*: PRT, prostate radiotherapy; ECOG, Eastern Cooperative Oncology Group; GS, Gleason score; iPSA, initial prostate-specific antigen.

**Fig 2:**
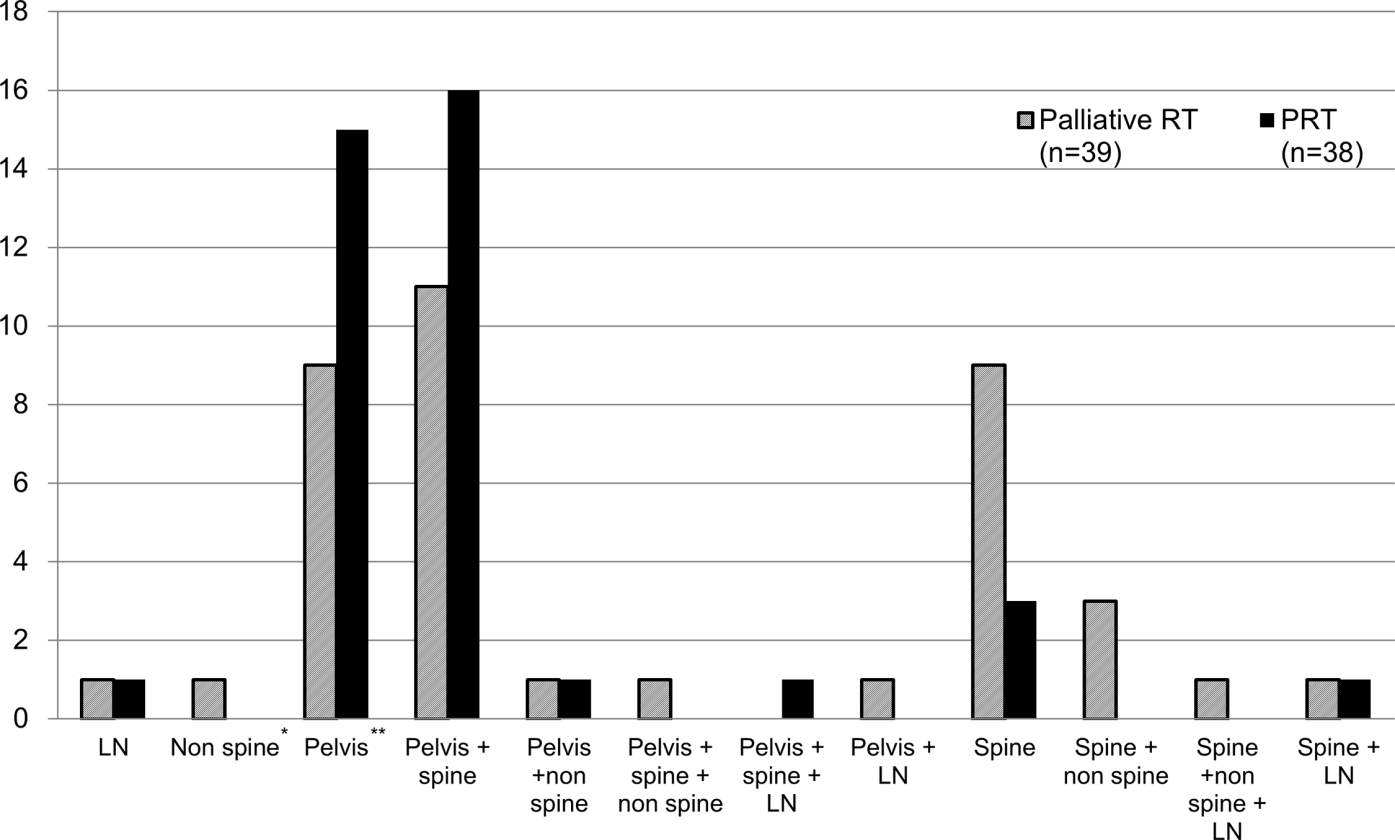
Extent and site of radiotherapy for patients receiving prostate radiotherapy (PRT) or palliative radiotherapy (RT). Non spine*: site of bone metastasis except in the pelvic bone and spine (C-T-L spine).Pelvis**: bone metastasis, including metastasis to the pubic, ischial, and iliac bones and the sacrum and proximal femur.

All patients received conventional or hypo-fractionated radiotherapy with a median dose of 60 Gy in 24 fractions (fx) (varying from 30 Gy/10 fx-72.6 Gy/33 fx) to the prostate (1.8–4 Gy per fraction). Common radiation schedules were 70 Gy/28fx (BED_3_ = 128.3 Gy) in 11 patients (29%) and 55 Gy/20fx (BED_3_ = 105.4 Gy) in 10 patients (26%). No patients received stereotactic body radiotherapy. Metastatic lesions received a median dose of 40 Gy in 10 fractions (range 22.5–54 Gy). Fifty-five (71%) patients were treated with tomotherapy, while others received conventional 3D-conformal radiotherapy. The extent and site of radiotherapy in the PRT and palliative radiotherapy groups are described in Fig 2.

The median nadir PSA level was relatively lower in the PRT group (1.12 vs. 0.61 ng/mL, *p* = 0.042) and more patients in the PRT group showed subclinical PSA levels (nadir PSA <4 ng/mL) than those in the non-PRT group (87% vs.55%, *p* = 0.002) (Table 1).

### Survival

**Fig 3:**
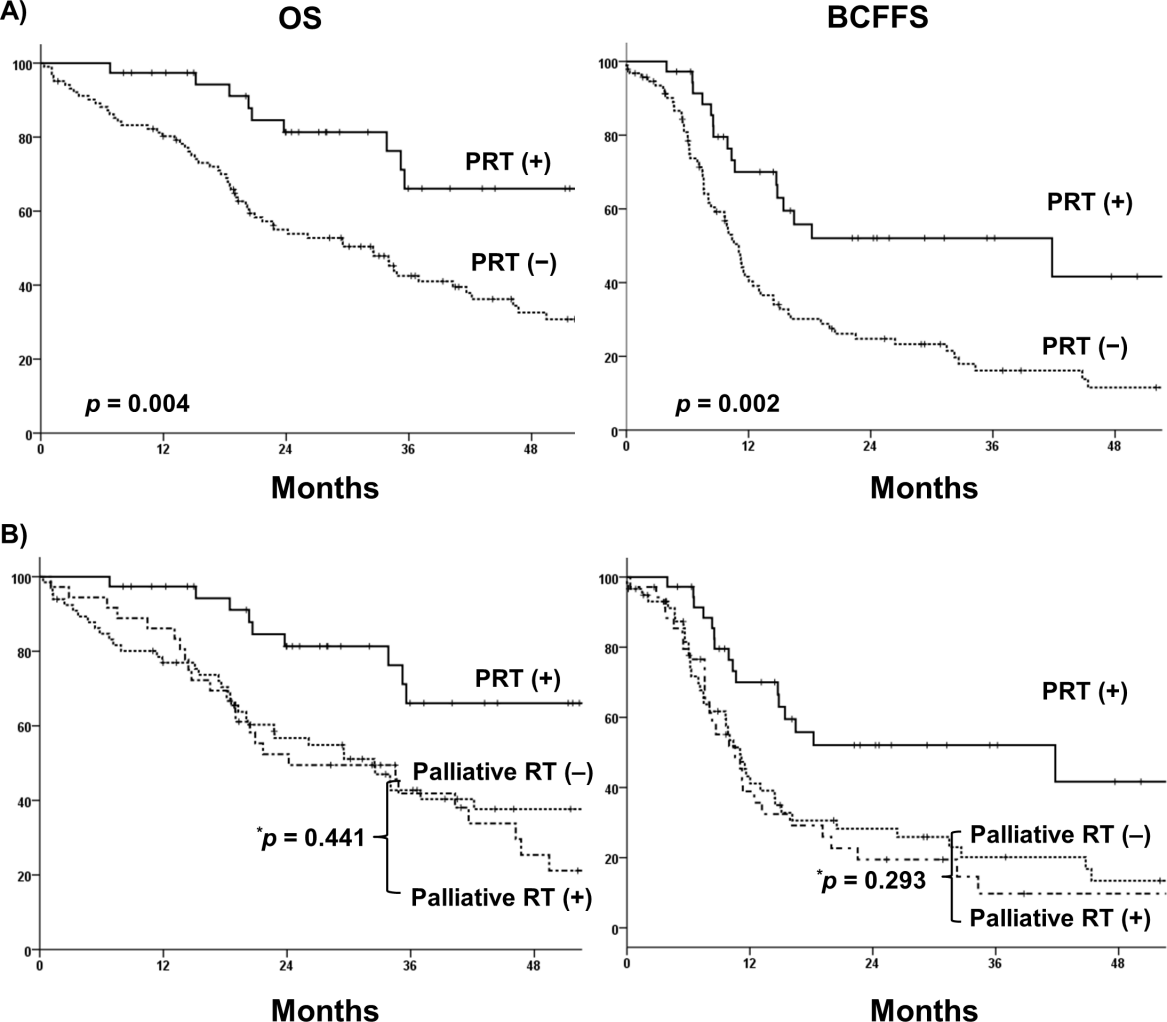
(A) Kaplan–Meier curves for overall survival (OS) and biochemical failure-free survival (BCFFS) of patients receiving prostate radiotherapy (PRT) and those who did not. OS (*p* = 0.004) and BCFFS (*p* = 0.002) were better in PRT patients than in non-PRT patients. (B) Kaplan-Meier curves for overall survival (OS) and biochemical failure-free survival (BCFFS) of non-prostate radiotherapy (PRT) patients with and without palliative radiotherapy (RT). In the non-PRT group, OS and BCFFS did not differ significantly between patients receiving palliative RT and those receiving non-palliative RT.

The median follow-up time was 34.0 months (range, 1.7–108.8 months) in the entire cohort. The 3-year OS of all patients was 48.2%, and BCFFS was 25%. The 3-year OS rate was higher in men receiving PRT than in those who did not (3-year OS: 69% vs. 43%, *p* = 0.004). Significantly prolonged BCFFS was also observed in the PRT group (3-year BCFFS: 52% vs. 16%, *p* = 0.002) (Fig 3A). Subgroup analysis was performed for the non-PRT patients. Of these, 39 patients received palliative radiotherapy to the metastatic sites with androgen deprivation treatment, whereas the others (n = 63) received systemic therapy only. No significant differences in OS (3-year: 50% vs. 40%, *p* = 0.441) and BCFFS (3-year 10% vs. 20%, *p* = 0.293) were observed between patients receiving palliative radiotherapy and those who did not (Fig 3B).

### Prognostic factors

Univariate analysis revealed that ECOG performance status, metastatic site, disease extent, and PRT were significant factors for OS. Furthermore, multivariate analysis confirmed irradiation to the prostate as a significant predictor of both OS (hazard ratio [HR] = 0.5, *p* = 0.046) (Table 2). For BCFFS, site of metastasis (bone vs. non-bone) and ECOG performance status scores (0–1 vs. 2–3), Gleason score and extent of disease did not show any significance (all *p* > 0.05), except PRT (*p* < 0.001) in univariate analysis.

**Table 2:** Univariate and multivariate analyses of overall survival.

Characteristics	Group	No	Univariate analysis		Multivariate analysis	
			3-year rate (%)	*p* value	HR	*p* value
Age (years)	<70	77	53	0.075		
	≥70	63	39			
ECOG status	0–1	111	65	0.004	1.57	0.121
	2–3	29	23			
GS	<8	22	41	0.758		
	≥8	104	54			
Metastasis site	Bone only	107	52	0.005	1.85	0.058
	Others	33	3			
Disease extent	1	25	57	0.007		0.096
	2–4	41	41			
	≥5	66	28			
	Visceral	8	0			
PRT	(+)	35	43	0.012	0.43	0.015
	(–)	105	62			

*Abbreviations*: PRT, prostate radiotherapy; ECOG, Eastern Cooperative Oncology Group; GS, Gleason score.

### Toxicities in PRT group

None of the 38 patients in the PRT group experienced severe (grade ≥3) GU or GI toxicity. In this group, 4 patients (11%) had grade 3 thrombocytopenia, and 3 (8%) had grade 3 leukocytopenia. Several patients received transfusion to prevent more severe adverse events, and most of the side effects were tolerable.

## Discussion

Our results from this study indicated that radiotherapy for the primary tumor improved prognosis of patients with metastatic prostate cancer. Men who received radiotherapy to the primary tumor had a favorable OS compared to those who did not (3-year OS: 69% vs. 43%, *p* = 0.004). Such improvement of prognosis was observed not only in OS but also in BCFFS (3-year BCFFS: 52% vs. 16%, *p* = 0.002). The effect of treatment was not significantly different among patients with various extent of metastasis. However, results of a stratified analysis suggested a greater effect among patients with only bone metastases at diagnosis and with good performance status.

Currently, radiotherapy for prostate cancer patients with distant metastasis is limited to palliative treatment to relieve local symptoms such as pain or urinary problems. Treatment of the primary tumor is often regarded inappropriate if metastasis is present. Moreover, Camphausen *et al*. revealed that the use of radiation to primary tumor resulted in the progression of previously dormant lung metastases by an imbalance of proangiogenic over antiangiogenic fartors in mice model [19]. However, in our study, radiotherapy to the primary prostate tumor did not impair survival but improved overall survival. This finding is consistent with previously reported findings of other malignancies, such as renal cell cancer, colorectal cancer, and breast cancer, for which a local treatment of primary tumor is effective in improving patients survival [2–5].

There is biologic evidence to support our proposal to treat primary tumor in patients with distant metastasis. Extensive experimental models had confirmed Paget’s original ‘‘seed and soil” theory that hypothesize the organ-preference of metastasis formation is the result of interactions between circulating tumor cells (the ‘‘seed”) and organ microenvironment (the ‘‘soil”)[20]. Not only supplying the “seed,” the primary tumor also has a major role to prepare the “soil”. Kaplan *et al*. reported that the initial event at a metastatic site is not the arrival of circulating tumor cells, but the recruitment of bone marrow-derived cells (BMDCs) at the metastatic site. These BMDCs make the microenvironment of the metastatic organ more acceptable to colonization of tumor cells forming ‘pre-metastatic niche’ [21]. Such a finding implicates that therapy directed toward the primary tumor, by inhibiting the endocrine molecules secreted by primary tumor, could delay the formation and growth of distant metastases. Weckermann *et al*. also evaluated the disseminated tumor cells (DTCs) before and after prostatectomy by analyzing the cytokeratin-positive (CK+) cells in bone marrow since CK+ cells were regarded as indicators of disseminated tumor cells (DTCs) [22]. They reported that DTCs before prostatectomy were significantly associated with an increased risk of metastases, but DTCs after surgery were not. This result suggested that the increased risk of metastasis manifested by DTCs was associated with an intact primary tumor and is consistent with the hypothesis that factors from the primary tumor are required to stimulate DTCs to colonize and grow.

A recent large-scale retrospective analysis also reports interesting findings. Positive pelvic lymph node is deemed to be a risk factor for distant metastasis and local therapy is in general omitted in favor of androgen-deprivation therapy. A study of the Munich Cancer Registry analyzed treatment outcomes in 938 prostate cancer patients with nodal metastases at the time of pelvic lymph node dissection [23]. Patients who underwent radical prostatectomy showed much favorable outcome compared to those who did not (10-year OS 64% vs. 28%). Although the group with radical prostatectomy had more favorable features, these findings showed at least a possible benefit from aggressive local treatment in patients with metastatic disease.

A possible hypothesis for treatment of the primary tumor could be the abscopal effect. However, this is less likely to happen after conventional fractionated RT alone, which may be insufficient to generate a systemic and robust antitumor effect. As initial treatment for metastatic prostate cancer or castration-resistant prostate cancer (CRPC), other systemic treatments as well as ADT could be administered. Docetaxel-based systemic chemotherapy has been generally used to treat metastatic prostate cancer after the approval of docetaxel in 2004 [24]. In addition, sipuleucel-T, an autologous prostatic acid phosphatase (PAP) directed cell-based immunotherapy product manufactured using patients’ own antigen-presenting cells, showed a survival benefit in a randomized phase III trial, and is recommended with category 1 evidence for patients who have a life expectancy of at least 3 months with a good performance status [25]. Cytotoxic T-lymphocyte antigen-4 (CTLA-4) blockade via the monoclonal antibody ipilimumab also has attracted attention due to positive results in patients with metastatic melanoma and renal cell carcinoma, as well as several other malignancies [26]. Ipilimumab is also currently being evaluated in the prechemotherapy and postchemotherapy settings in men with metastatic CRPC. Further evidence of the efficacy of combined modalities for metastatic prostate cancer is needed so that systemic control and a radiation-induced immune response can be amplified sufficiently.

Another practical reason for us to suggest primary tumor radiotherapy for stage IV prostate cancer is that recently developed techniques have significantly reduced treatment-related side effects. In other words, addition of the primary tumor in the radiotherapy field does not cause patient discomfort. In this study, 71% of patients in the PRT group received intensity-modulated radiotherapy (IMRT) using helical tomotherapy and no patient experienced severe (grade ≥3) GI or GU toxicity. The IMRT is a newer radiotherapy technique that uses intensity-modulated beams to provide multiple intensities, allowing more concave dose distribution than conventional techniques. Furthermore, the chronic and acute toxicities associated with radiotherapy for prostate cancer are well documented [27]. In the era of IMRT, several studies have demonstrated the feasibility and efficacy of IMRT for prostate cancer in a large number of patients. Acute and late GI and GU toxicities seem to be significantly lower than those observed with conventional 3D-conformal radiotherapy techniques [15]. Moreover, IMRT using helical tomotherapy makes it possible to treat a wide range of lesions including the prostate and metastatic sites simultaneously. Compared to other local treatment such as prostatectomy. Based on a risk versus benefit analysis, radiotherapy with this less invasive and more effective treatment technique is suitable for prostate cancer patients with distant metastases, compared to other local treatments such as prostatectomy.

However, IMRT using helical tomotherapy could treat a wide range of metastatic lesion which is spreading vertical axes and most common acute side effect in PRT group was thrombocytopenia and leukocytopenia. Previous report in our institution suggested that the tolerance cutoff point of red marrow was 26.8% to avoid severe leukocytopenia (grade ≥3) [28]. With this finding, we have taken into account for the proportion of bone marrow when we treat wide range of bone metastasis.

In this study, good performance status, bone only metastasis and limited disease extent were identified as prognostic factors for OS (ECOG PS 0–1 vs. 2–3, 3-yr OS 65% vs. 23%, *p* = 0.004; bone only metastasis vs. others, 3-yr OS 52% vs. 3%, *p* = 0.005; disease extent, single metastasis vs. 2–4 metastases vs. ≥5 metastases 3-yr OS 57% vs. 41% vs. 28%, respectively, *p* = 0.007). Our results showing a better prognosis in patients with bone-only metastasis are consistent with observations from a recent study of patients from the SEER database. This study reported that men with visceral metastasis showed inferior OS to that of men with bone-only metastasis [29]. These findings suggested that prostate may have several phenotypes that predispose to different natural histories when survival represents the end point. While the underlying mechanism of different survival outcome according to the metastatic site observed between studies unclear, we emphasize that retrospective studies should be cautiously interpreted within their limitations.

The association with limited disease extent and survival benefit in metastatic disease has been studied in other tumors including breast cancer, renal cell carcinoma and colorectal cancer [4,30,31]. Nguyen *et al*. revealed that limited metastatic disease, defined as <5 metastatic lesions is a favorable prognostic factor in stage IV breast cancer (5-yr OS 29.7% vs. 13.1%, *p* <0.001). They also found that locoregional treatment for breast cancer improved overall survival (5-yr OS 35.5% vs. 20.4%, *p* = 0.01). These results are in accordance with our study. Prognostic factors defined in this study could help the clinicians identify reasonable candidates for radiotherapy to primary tumor and evaluate appropriate treatment strategies. Selected patients with limited extent of bone metastasis in good performance status could obtain the maximum benefit from this treatment strategy.

As the present investigation is not a randomized study, it is important to consider the possibility of selection bias due to unrecorded factors. In such a case, the differences in mortality risk observed between patients receiving PRT and those who did not could reflect the lower utilization of PRT in men with putative poorer disease prognosis. In addition, further evaluations including metastatic markers or immunohistochemical staining results for understanding the biologic behavior of tumors and metastatic sites are needed. Therefore the results of this pilot study warrant a subsequent prospective randomized control trial to find out the effectiveness of aggressive treatment to primary prostate gland in patients with stage IV prostate cancer. The STAMPEDE (the Systemic Therapy in Advancing or Metastatic Prostate Cancer: Evaluation of Drug Efficacy) trial from Medical Research Council Clinical Trials Unit (MRC CTU) was initiated in 2013 and result from this trial could prove the benefit of PRT [32].

## Conclusions

Radiotherapy including treatment of the primary tumor for prostate cancer patients with distant metastasis resulted in improved biochemical control and long-term survival. The results of this study warrant a randomized controlled clinical trial to confirm these.
